# Illusory deformation of the finger is more extensive in the distal than the lateral direction

**DOI:** 10.1177/20416695241254526

**Published:** 2024-05-15

**Authors:** Yutaro Sato, Godai Saito, Kenri Kodaka

**Affiliations:** 12963 Nagoya City University, Japan; 13101 Tohoku University, Japan; 12963 Nagoya City University, Japan

**Keywords:** double-touch operation, illusory finger deformation, proprioceptive drift, sense of ownership

## Abstract

Previous studies have examined the rubber hand illusion with finger lengthening, but there is limited research on finger widening. This suggests a strong cognitive bias toward the illusory expansion of the finger in a distal direction rather than lateral. To test this, we compared the illusory deformability of the finger in the distal and lateral directions through the generation of illusory finger deformation using a double-touch operation, referred to as the numbness illusion. Our results showed that perceived distal distortion was wholly superior to perceived lateral distortion in terms of sense of ownership ratings. Moreover, the extent of the perceived deformation was greater in the distal than lateral direction, supporting our hypothesis that there is a distal bias. We suggest that this preference may be because the presence of multiple joints is required to create illusory deformation in the target direction.

In artistic depictions of the body, a dynamic stretching of the arms or fingers is often observed (e.g., Monkey D. Luffy in *One Piece* and King Piccolo in *Dragon Ball*). Does this trope have a basis in susceptibility to body-ownership illusions? This anisotropic tendency is also evident in psychological studies that employ the rubber hand illusion (RHI). Bodily illusion studies highlight the flexibility of the mental representations of the hand when multisensory correlated integration is utilized (Blanke, 2012; Botvinick & Cohen, 1998). Broadly speaking, research into illusory body deformation falls into two types: whether the illusory body is geometrically similar to the original (e.g., the Enlarged fake hand illusion: Giurgola et al., 2021; Pavani & Zampini, 2007; the Barbie doll illusion: Van Der Hoort et al., 2011) or not. Most illusory body deformations in which this is not the case occur in a proximal-to-distal direction, as when the arms or fingers are stretched in the direction of limb growth (e.g., MIRAGE: Hansford et al., 2023, 2024; Newport & Preston, 2010; Stretchar(m): Kodaka & Mori, 2017; the elastic legs illusion: Kodaka et al., 2020). However, to the best of our knowledge, research into illusory deformation in a medial-to-lateral direction, leading to the widening of the arm, hand, or finger has almost not been performed using the RHI. Although this trend may simply be derived from an interest in functional improvements in the extension of the limbs or fingers, it is nevertheless worth examining whether there is greater elasticity in perceptions of illusory deformations in the distal than in the lateral direction. This proposition seems to find support in related studies. Tactile acuity is reported to be higher across than along the hand (Cody et al., 2008; Longo, 2022), and the ownership illusion is more adversely affected by visuo-tactile incongruence when it occurs in the medial-to-lateral direction than the proximal-to-distal direction of the hand (Seo et al., 2022). Thus, we hypothesize that this difference in the number of reports on the two directions reflects a cognitive bias toward greater elasticity in perceived illusory body deformation along the distal direction. This idea is not self-evident in real-life physical experiences since a paralyzed finger is generally felt as thicker/wider than the longer finger (Walsh et al., 2015), which is counter to our hypothesis. Therefore, in this study, we endeavor to identify which direction is more subject to illusory deformation, the proximal-to-distal or medial-to-lateral axis.

To assess our hypothesis, a comparison of the distal and lateral spatial limits of the RHI is an appropriate means of examining the anisotropy of the illusory hand's mobility. However, this approach would not be sufficient alone, because the standard layout of RHI does not specifically yield the illusory hand's deformation. This is because it is easy to fill the spatial gap between the fake and real hand solely by illusory motion with proprioceptive rotation of the shoulder and elbow (the proprioceptive drift of whole hand). In the illusion, in altering the proprioception of the arm, there is no need to go induce a bodily deformation with high cognitive costs that cannot occur in reality. This may be a reason for the limited number of studies on illusory finger deformation in the classical RHI. In fact, the spatial limit for RHI, measured as the distance between the physical and rubber hand in cases where illusory ownership of the rubber hand is significantly reduced (Aimola Davies et al., 2013; Erro et al., 2020; Lloyd, 2007), is relatively close to the hand's peri-personal space (PPS), which represents the spatial boundary around the body in which the physical hand, with or without a tool, can potentially reach (Brozzoli et al., 2011; Rizzolatti et al., 1981; Serino et al., 2015). This relationship indicates that classic RHI, in which the distortion of the mental representation of the body mainly occurs with the proprioceptive drift of the arm, would not be appropriate for the identification of the spatial boundary of illusory finger deformation, regardless of the axis of the deformation. From these considerations, this study explores the alternative approach for inducing a selective proprioceptive drift in a specific finger joint while restraining the proprioceptive rotation of the arm.

Here, we utilize the numbness illusion (NI) as an ownership distortion that involves selective proprioceptive drift (Dieguez et al., 2009). The NI induces an illusory numbness in another person's finger experienced as one's own by touching one's own finger and another person's at the same time (Figure 1). This illusory numbness is a consequence of the absence of anticipated somatosensory stimulation from an object to which ownership is temporarily given (Aymerich-Franch et al., 2016). Importantly, Dieguez et al. (2009) discovered that the NI produces both numbness and the illusory sense of finger widening. This suggests that ownership is projected over an extended area that includes both fingers. For two reasons, the NI is considered a variant of the RHI. First, the double-touch of both fingers must be simultaneous, which corresponds to the procedure used in the RHI (Blanke, 2012). Second, the difference between the NI and the nonvisual somatic RHI (Ehrsson et al., 2005) is the person who touches the participant's hand, the participant (NI), or the experimenter (somatic RHI), as shown in Figure 1. The self-touch, specific to the NI, strongly anchors the mental representation of the finger to the original physical location, inhibiting the free proprioceptive drift of both the touched point and the body parts located more proximal than the touched point. This includes the wrist, elbow, and shoulder. It is speculated that this proprioceptive inhibition is essential in yielding the illusory feeling of the finger widening in the NI. We expect the NI's capacity to produce the illusory finger's deformation to apply to multiple axes of the finger. In this study, we clarify which axis is more deformable in the NI. Specifically, the spatial boundary of the illusory finger deformation is examined for the distal layout, where the experimenter's finger is placed on the extension of the participant's finger, and the lateral layout, where the participant's and experimenter's fingers are placed parallel to one another (Figure 1). The NI also shows a promising prospect for predicting the spatial cognitive boundary of the deformed finger for each axis in a neutral manner because the NI also appears when the eyes are closed. Within this non-visual approach, the subjective shape of the deformation of the finger must be cognitively self-organized in the absence of top-down visual information. This characteristic is useful for handling the potentially diverse mental representation of the hand (Kodaka & Ishihara, 2014).

**Figure 1.**
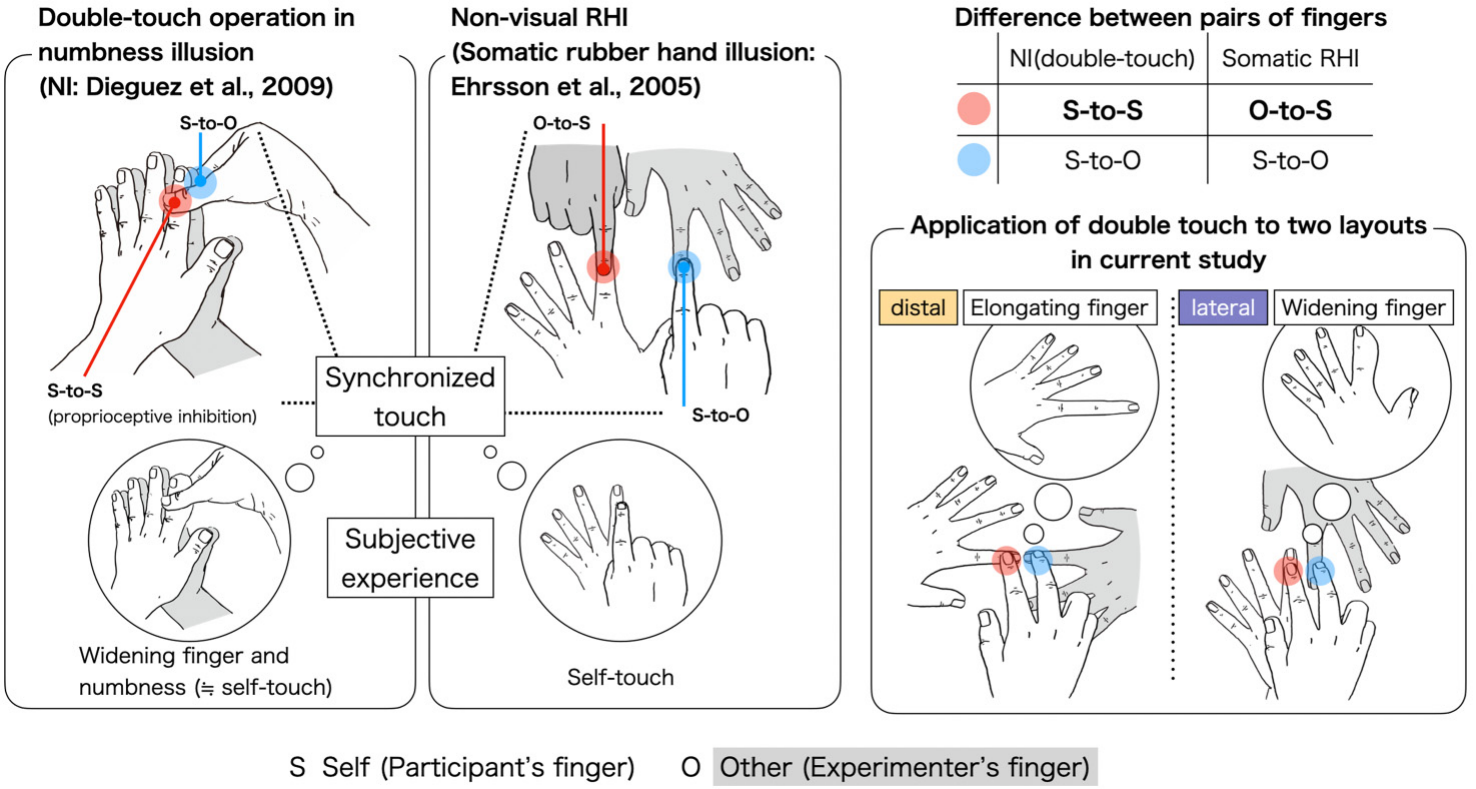
Various hand layouts for the numbness illusion (NI) and the somatic rubber hand illusion (somatic RHI). The sole distinction between these illusions is whether the experimenter (somatic RHI) or the participant (NI) touches the participant's hand. In this study, the double-touch operation was adopted for the distal and lateral directions of the finger, as shown in the illustration on the right side of Figure 1.

In our previous experiment, as described in the Supplemental material, only subjective evaluations of the illusion (i.e., of the sense of ownership and sense of finger deformation in terms of length or width) were conducted among two receptive finger layouts (the distal and the lateral). The result supports the hypothesis that longer finger deformations are more likely to occur than wider deformations. In this study, we examined the behavioral index, developing the body landmark task (Longo & Haggard, 2010) to measure the transition of the position of the target finger during the illusion, as well as the subjective evaluations of the illusion. The overall results support our hypothesis, both in terms of subjective evaluation and behavioral assessment.

## Methods

A total of 25 healthy students (14 female; with a mean age of 20.6 years, *SD *= 1.85, age range = 20–29) participated in the study. We used the Japanese version of the Flinders Handedness Survey (Okubo et al., 2014) to determine the participant's handedness, finding that 23 were right-handed (*M *= 9.74, *SD *= 0.69), 1 was left-handed (*M *= –10.0, *SD *= 0), and 1 was ambidextrous (*M *= 2.0, *SD *= 0). All participants received a book of tokens worth 1000 yen as compensation. The study was approved by the Ethics Committee of the Nagoya City University and conducted in accordance with the 1964 Declaration of Helsinki and its subsequent revisions. Informed consent to participation and publication was obtained from all of the 25 students in written form before they took part in the study. Using statistical calculator Morepower 6.0 (Campbell & Thompson, 2012), we calculated the necessary sample size for a 2 × 2 within-participants design: an effect size based on the main effect of layout in our previous research based on subjective evaluation (*n* = 25), $\eta_{p}^{2}$ = 0.27; a power of 0.8; an alpha of 0.05. This yields a total sample size of 24 participants, so our sample size of 25 participants was well powered. The effect size ( $\eta_{p}^{2}$ = 0.27) was seen in the layout effect comparing 3 and 6 cm and excluding the other three conditions (2, 4, and 5 cm) in the previous study reported in Supplemental material.

### Subjective Evaluation Sessions

**Figure 2.**
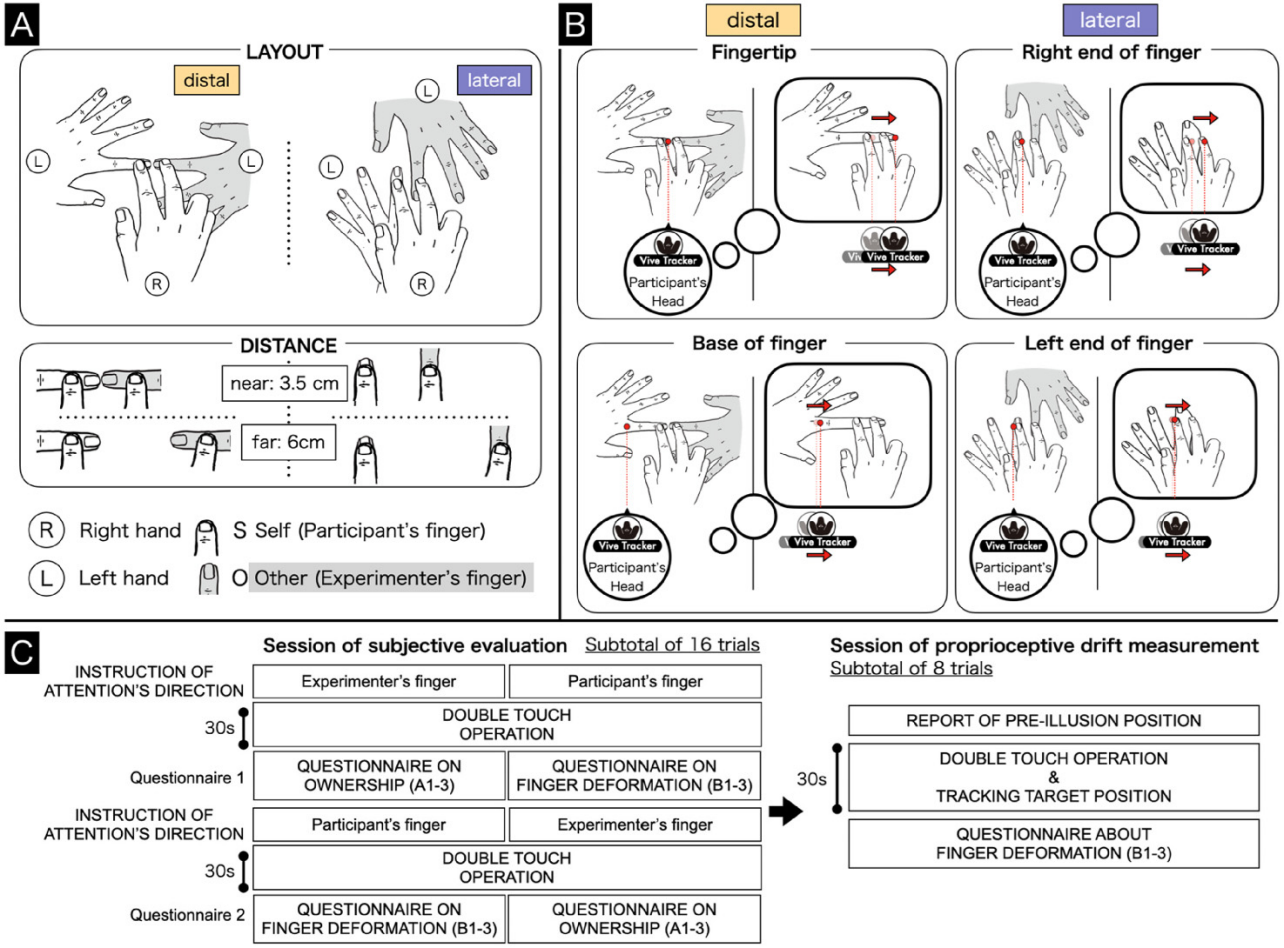
(A) The double-touch operation was conducted in two layouts and two distance conditions. (B) The procedure used in proprioception measurement sessions, which involved tracking the receptive fingertip and base of the receptive finger in the distal layout, and the right and left sides of the receptive finger in the lateral layout. The red arrows correspond to the typical proprioceptive drift. (C) The experimental procedure, which comprised a total of 24 trials (the subjective evaluation task: 2 layout conditions × 2 distances conditions × 2 trials × 2 types of statements A and B; the drift measurement: 2 distances × 4 targets). For each layout condition, the order was varied between participants in a counter-balanced manner (distal-to-lateral or lateral-to-distal; near-to-far or far-to-near).

The participant sat opposite the experimenter with their right (administrative) hand on the armrest attached to the desk. There were two finger layout conditions: distal and lateral. In both layouts, the participant was asked to place their left (receptive) hand on the desk with the palm down. In the distal layout, the fingers faced right; in the lateral layout, the fingers were upright (Figure 2A). The experimenter's left (receptive) hand was placed in an identical position to that of the participant, with the index fingers of the two placed close together in opposite directions in the distal layout or beside each other with a fixed gap in between them in the lateral layout. In both layouts, the double-touch procedure was conducted with the index and middle finger of the administrative hand with the participant blindfolded. The distance between the two administrative fingers was 3.5 or 6 cm (Figure 2A). The instructions for the double-touch required the touch to be to the first joint of the two receptive fingers, avoiding the fingernails.

**Figure 3.**
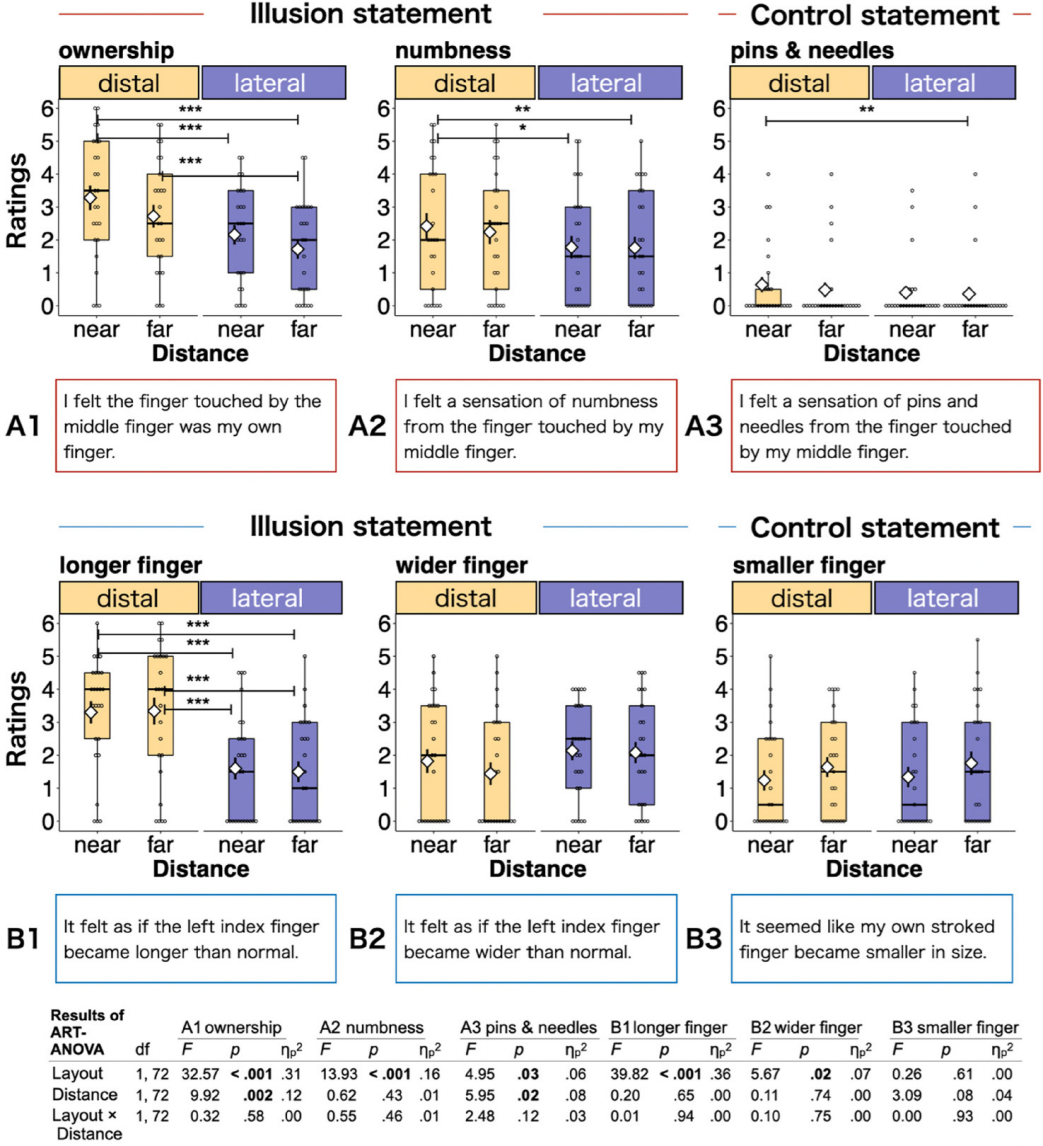
The average values for the participants’ subjective evaluations of ownership, numbness, and deformation in the double-touch operation, with comparative control values. The bold lines indicate the medians and the white diamonds indicate the means. The error bars show the standard error. The upper and lower limits of the box plots are the 75th and 25th percentiles. Asterisks indicate significant differences between comparisons (∗*p *< .05, ∗∗*p *< .01, ∗∗∗*p *< .001). The table in the bottom panel displays the ART ANOVA results.

In each experimental condition, participants were instructed to rhythmically oscillate two receptive fingers by their index and middle fingers, maintaining contact with both their own fingers and that of the experimenter (the double-touch operation). This task was performed while maintaining uniform pressure on the receptive fingers for 30 s, all while listening to white noise through headphones to mask any extraneous sounds. The oscillation direction was to be perpendicular to the alignment of the receiving fingers (distal layout: anterior-posterior, lateral layout: left-right). The oscillation frequency was set at 2 Hz, and participants were required to maintain this consistent rhythm by imitating the oscillations taught by the experimenter prior to the task. Each trial began after the instructions regarding the target of the participant's attention had been given. In task A, participants were instructed to focus on their perception of the sensation from the experimenter's receptive finger. Conversely, in task B, participants were asked to direct their attention toward their perception of the size of their own receptive finger. Following each trial, participants were immediately required to provide their responses to a subjective evaluation using a 7-point Likert scale. On this scale, a rating of 0 indicated “completely disagree,” a rating of 3, “neither agree nor disagree,” and a rating of 6, “completely agree” for three distinct categories of statements. Specifically, in task A, the illusion statements were concerned with ownership (A1) and numbness (A2), while the control statements were about pins and needles (A3). In task B, the illusion statements were concerned with length deformation (B1) and width deformation (B2), while the control statement was about smaller finger deformation in size (B3) as shown in Figure 3. The detail of experimental procedure is shown in Figure 2C.

### Proprioceptive Drift Measurement Sessions

To determine subjective illusory finger deformation distances, measurements of proprioceptive drift were performed for four distinct finger positions (Figure 2B). To assess perceived finger length in the distal layout, proprioceptive measurements were taken for both the fingertip and the base of the receptive finger. To assess perceived finger width in the lateral layout, proprioceptive measurements were taken around the first joint for both the left and right sides of the receptive finger. In the body landmark task (Longo & Haggard, 2010), participants used a long metal baton to indicate the perceived positions of the targets, such as the base of the fingers or the fingertips. In this measurement, participants cannot point to the perceived position using the hand due to the double-touch operation. As an alternative measurement, the participants wore a headband with a Vive Tracker 3.0 (HTC) device throughout the session that was positioned at the center of their forehead, where the participants align the center of their forehead horizontally with the perceived target finger part during a 30-s double-touch operation. We assume that the results for the subjective finger deformation produce a stronger proprioceptive drift in the fingertip or the right end of the receptive finger and a weaker drift in the base or the left end of the receptive finger.

At the pre-illusion stage, the blindfolded participant was asked to pay attention to the target position when their finger was touched by the experimenter. They were then instructed to align the center of their forehead with the designated target position and verbally affirm “Yes” to indicate that their head was correctly positioned where they feel the target. Following the pre-illusion stage, participants were instructed to maintain head tracking on the perceived finger position while engaged in the 30-s double-touch procedure and listening to white noise, as in the subjective evaluation sessions. In the above procedure, the participants kept their eyes closed. Afterward, participants were immediately required to provide ratings on the illusory finger deformation statements (“B1” to “B3”). This session comprised a total of eight trials to incorporate tracking of two positions within the distal and lateral layouts as shown in Figure 2C.

## Results

### Data Analysis

A QQ plot inspection was performed to assess data distribution. Due to the residuals of the ANOVAs for the subjective evaluation scores and deformation distances were non-normally distributed, we performed nonparametric analysis using a two-way repeated-measure ART ANOVA (analysis of variance with an aligned rank transformation; Wobbrock et al., 2011). We also conducted nonparametric pairwise comparisons using the ART–C procedure (Elkin et al., 2021), with Bonferroni correction of each condition. The statistical analyses were performed using R version 4.2.2 (R Core Team, 2022). Statistical significance was set at *p *< .05.

### Subjective Evaluations

First, a two-way repeated-measures ART ANOVA (layout × distance) was conducted for subjective rating for each six statements (A1–3 and B1–3). The analysis showed significant main effects for the layout for ownership (*F*(1, 72) = 32.57, *p *< .001, $\eta_{p}^{2}$ = 0.31), numbness (*F*(1, 72) = 13.93, *p *< .001, $\eta_{p}^{2}$ = 0.16), pins and needles (*F*(1, 72) = 4.95, *p *= .03, $\eta_{p}^{2}$ = 0.06), longer finger (*F*(1, 72) = 39.82, *p *< .001, $\eta_{p}^{2}$ = 0.36), and wider finger (*F*(1, 72) = 5.67, *p *= .02, $\eta_{p}^{2}$ = 0.07), but not for the control statements of B3. The analysis also found significant main effects of distance for ownership (*F*(1, 72) = 9.92, *p *= .002, $\eta_{p}^{2}$ = 0.12) and pins and needles (*F*(1, 72) = 5.95, *p *= .02, $\eta_{p}^{2}$ = 0.08), although this was not found for the other statements. In addition, no significant interactions were found for all statements. The layout effects for statements A1 and A2 showed a higher contribution to yielding the illusion in the distal layout relative to the lateral layout, while those for statements B1 and B2 showed the distal/lateral layout contributes to yielding the longer/wider finger's deformation relative to the other layout. The distance effect in A1 indicates that the double touch at a closer distance contributes to the yield of a stronger ownership illusion, aligning with the general theory of the RHI.

Second, pairwise comparisons were conducted for each of the six pairs of conditions for all statements. For ownership and numbness, the comparison indicated that the distal-near layout yielded higher ratings for ownership and numbness than the two conditions in the lateral layout, and the distal-far layout yielded significantly higher ratings for ownership compared to the lateral-far layout (all *p *< .05). For the statement concerning the longer finger, the comparison found that each of the two conditions in the distal layout produced significantly higher ratings than the two conditions in the lateral layout (all *p* < .001). The statistical details are given in the boxplot in Figure 3 and the table in the bottom panel of Figure 3.

### Subjective Finger Deformation Distance

**Figure 4.**
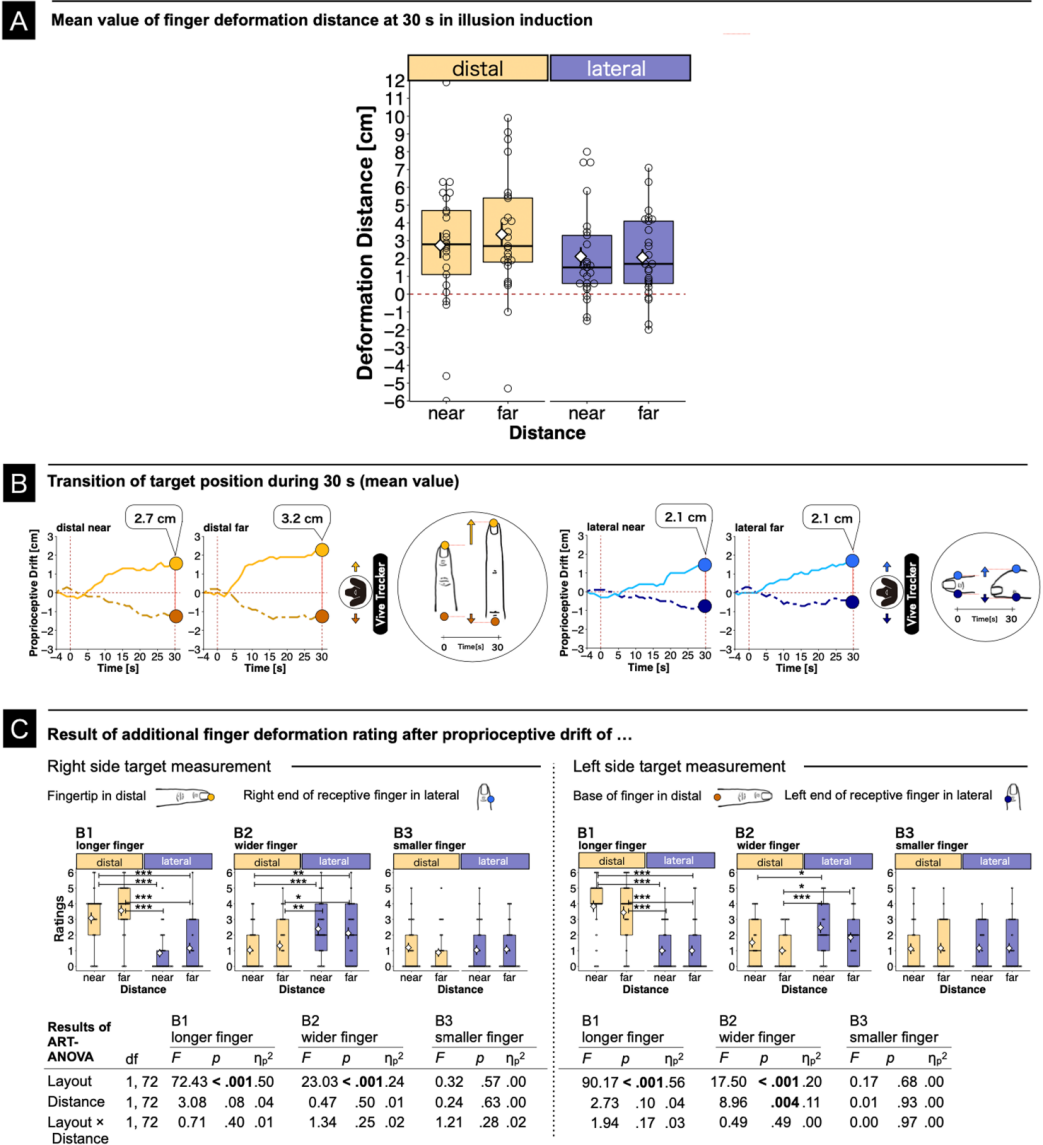
(A) Perceived finger deformation distances in the double-touch operation for deformation in the distal and lateral directions. The box plot depicts the finger deformation distance. The table in the right panel displays the ART ANOVA results. (B) The plot shows the mean transition values of the target position over 30 s. The dots indicate body part positions: yellow, fingertip; brown, base of finger; light blue, right side of finger; deep blue, left side of finger. Each position was measured separately. (C) Result of additional agreement rating after finger deformation measurement (B1–B3). The result of proprioceptive drift was reported in main text. The box plot depicts the mean subjective ratings for deformation statements. The table in the bottom panel shows the ART ANOVA results. (Box plots in A and C) Bold lines indicate the medians and white diamonds indicate the means. Error bars indicate the standard errors. The upper and lower limits of the box plots indicate the 75th and 25th percentiles. Asterisks indicate significant differences between comparisons (∗*p *< .05, ∗∗*p *< .01, ∗∗∗*p *< .001).

Figure 4A shows the result for distances of illusory finger deformation using a boxplot. Figure 4B shows the mean transition of the target position over 30 s in the proprioceptive drift measurement session. Note that the deformation distance in Figure 4A corresponds to the transition that is observed at the end of the 30-s session in Figure 4B. The deformation distances are defined as the differences in the perceived locations of the target positions such as the receptive fingertip and the base of the receptive finger in the length deformation and between the right and left sides of the receptive finger in the finger width deformation. The deformation distances were analyzed using a two-way repeated-measures ART ANOVA (layout × distance). In this analysis, a significant main effect was found for the layout (*F*(1, 72) = 7.87, *p *= .006, $\eta_{p}^{2}$ = 0.10), while the main effect of the distance and the interaction between the two factors were not significant (distance: *F*(1, 72) = 0.36, *p *= .55, $\eta_{p}^{2}$ = 0.00; layout × distance: *F*(1, 72) = 0.06, *p *= .81, $\eta_{p}^{2}$ = 0.00). The layout effect showed that the distal layout yielded the stronger deformation than the lateral layout.

We analyzed the same subjective finger deformation ratings (B1 to B3) again after each experimental task for drift measurements (right side target measurement: the fingertip and the right side of the receptive finger; left side target measurement: the base and the left side of the receptive finger). The results showed significant main layout effects for both the length and width of the finger on both sides of the drift measurement. A main distance effect was found only for the base and the left side of the receptive finger. The pairwise comparisons found significant differences in the length and width on both sides of the drift measurements. The statistical details are shown in Figure 4C.

## Discussion

The present study measured the effects of the layout (namely, the direction of illusory finger deformation) and distance in illusory finger deformation using the double-touch procedure. Our hypothesis regarding the anisotropy of illusory finger deformation was supported by the significant effect of the direction of finger deformation on the subjective evaluation of finger ownership. More importantly, significantly higher ratings were observed for the distal than the lateral layout, even when the distances were the same, which replicates the findings of our previous study featuring subjective evaluation. Our hypothesis was also confirmed by the behavioral measurement, where the effect of finger deformation direction was significant for the illusory distance. In summary, these results support the notion that the perception of illusory finger deformation exhibits greater elasticity in the distal than in the lateral direction.

The observed significant effect of distance on the subjective evaluation of finger ownership suggests that the deformable boundary (DB) of the finger is likely situated within the range of 3.5–6.0 cm. Notably, the observed DB was far less than the spatial boundary usually measured in the RHI (over 20 cm: Lloyd, 2007). This critical difference can largely be attributed to the proprioceptive inhibition of the proximate body parts (the shoulder, elbow, and wrist) caused by self-touch, which allows farther hand reach even with slight rotation as long as it is freely operational. This effect is corroborated by studies that indicate that engaging in active self-touch enhances the precision of the proprioceptive representation of the touched body part (Cataldo et al., 2024). Without the proprioceptive distortion of such proximate joints, deforming the receptive finger is the only means of filling the spatial gap between administrative (self-to-other) touch and receptive (self-to-self) touch. Deformation of a specific finger is cognitively more costly than rotating the shoulder, elbow, and wrist, regardless of whether it occurs in physical or mental space. Thus, the decline in the PPS boundary is clearly attributable to the proprioceptive inhibition caused by self-touch during the double-touch procedure.

Although this form of proprioceptive inhibition works regardless of the double-touch direction, the ownership ratings and finger deformation distances were greater in the distal than the lateral direction. What forms such anisotropic deformability? Our hypothesis is that the number of joints that fall along the deformable axis is a critical factor in yielding this anisotropy. Joints are referred to as “perceptual anchor points,” where tactile resolution is enhanced, contributing to improved proprioceptive guidance for recognition of the mental representation of the finger (Cody et al., 2008; De Vignemont et al., 2009). Thus, it is reasonable to assume that altering the proprioception of a specific joint forms the main factor in altering the subjective length of the limb or the finger. Our hypothesis is based on the assumption that the illusory deformation of the body part occurs as drift in a specific joint, with the remaining joints anchored to the original location, extending the distance between the anchored joint and the drifted joint. Consequently, the presence of multiple joints is necessary for the illusory deformation in the target direction. In our experiment, the self-touch was given at a point around the first joint of the finger, irrespective of the layout. When the double-touch was performed in the distal direction, the proprioceptive drift was applied to the first finger joint, with the second and third joints blocked and prevented from also moving in the distal direction. In the lateral layout, the first finger joint was affected both by proprioceptive drift and proprioceptive inhibition, because there was only a single joint in the lateral direction. Therefore, it is speculated that, in double-touch in the lateral direction, proprioceptive drift and inhibition are competing to affect the single joint. This results in poorer deformation. Importantly, this concept also applies to the deformation of other body parts beyond finger. It predicts that deformation anisotropy will be observed in the arm and leg but not in the palm and the back of the hand.

Finally, our study had some limitations. First, our assertion regarding the elasticity of deformations was based strictly on absolute magnitudes of distance; a separate discussion that considers proportional distances is required. In fact, the predominant direction of elasticity might potentially be reversed if the proportional distances were used, as the change ratio in localized numbness effects on the perceived width of the index finger is greater than that for the perceived length (+30% vs. 5%) (Walsh et al., 2015). A comparison of the values obtained for the different finger lengths (e.g., index finger vs. thumb) could provide valuable insights into the impact of proportion. Second, it is unclear how far the high-level effect of body representation is related to this anisotropic effect. While we do not experience longer finger deformations in daily life, we can easily imagine that they would serve a useful function through increasing the range of interaction between the body and the environment. On the other hand, wider finger deformations would tend to impair body mobility, as experienced in the case of swelling. This could be a major reason why there has been limited research on illusions involving wider body parts. However, in spite of this, several studies have reported illusions of changes in overall body size (Giurgola et al., 2021; Pavani & Zampini, 2007), indicating that wider body parts may be acceptable where the proportion of the body part is not distorted. Thus, investigating the implications of the varied potential functionality that is associated with deformation poses an intriguing challenge. Third, it is unclear whether the anisotropic effect is enhanced for children, who are reported to have high plasticity in their body representations (Cowie et al., 2022). Addressing these issues remains a significant challenge.

## Supplemental Material

sj-pdf-1-ipe-10.1177_20416695241254526 - Supplemental material for Illusory deformation of the finger is more extensive in the distal than the lateral direction Supplemental material, sj-pdf-1-ipe-10.1177_20416695241254526 for Illusory deformation of the finger is more extensive in the distal than the lateral direction by Yutaro Sato, Godai Saito and Kenri Kodaka in i-Perception
