# Supplementary material for: Illusory deformation of the finger is more extensive in the distal than the lateral direction
Source: Iperception. 2024 May 15;15(3):20416695241254526. doi: 10.1177/20416695241254526 (PMC11100397; doi:10.1177/20416695241254526)
Supplement: sj-pdf-1-ipe-10.1177_20416695241254526 - Supplemental material for Illusory deformation of the finger is more extensive in the distal than the lateral direction [file sj-pdf-1-ipe-10.1177_20416695241254526.pdf]

Supplemental data: Illusory Deformation of the Finger Is More Extensive in the Distal than the Lateral Direction

Yutaro Sato[1], Godai Saito[2], and Kenri Kodaka[1]
[1] Nagoya City University, Nagoya, Aichi, Japan
[2] Tohoku University, Sendai, Japan

(1) Previous study based on subjective evaluation

1.1 A-priori power analysis

Using statistical calculator, Morepower 6.0 (Campbell & Thompson, 2012), we calculated the necessary sample size for a 2 × 5 within-participants design: an effect size based on the main effect of layout in pilot research (n = 9), η_p^2 = .27; a power of 0.8; an alpha of 0.05. This yields a total sample size of 24 participants, our sample size of 26 participants was well powered.

1.2 Methods

A total of 26 healthy students (15 females, two left-handed and one ambidextrous which is based on participant's self-report; *M* = 21.5 years, age range = 20–24) participated in the study. All participants received a book of tokens as compensation (1000 yen). The study was approved by the Ethics Committee of the Nagoya City University and conducted in accordance with the Declaration of Helsinki.

The participant sat opposite the experimenter with their right (administrative) hand resting on the armrest. There were two conditions concerning the finger layout: distal and lateral. In both layouts, the participant was asked to place their left (receptive) hand on the desk with the palm down. They had their fingers facing right in distal layout or with the fingers upright in the lateral layout (Figure 1 left). The experimenter's left (receptive) hand was placed identically to the participant, where the two index fingers were placed close together in opposing directions in the distal layout or were placed beside each other with a fixed gap in between them in the lateral layout. In both layouts, the double-touch operation was conducted

Corresponding author:
Yutaro Sato, Nagoya City University, 2-1-10, Kita Chikusa, Nagoya, Aichi 464-0083, Japan.
Email: sato12yutaro@gmail.com

with the index and middle finger of the administrative hand with eyes blinded. The distance between the two administrative fingers was 2, 3, 4, 5, or 6 cm (out of five distance conditions). In addition, the double-touch was asked to provide around the first joint of the two receptive fingers with keeping away from their fingernails. Consequently, only in the 2 cm distal layout, the experimenter's left index finger was replaced with the little finger placed orthogonally to the participant's index finger, which was due to prevent mutual overlap, as shown in Figure 1. In each condition, the participants tapped the two receptive fingers for 30 seconds, where they were required to match the speed of the double-touch with the metronome (2 Hz). Figure 2A show example of an experimental procedure (4cm condition in distal and lateral layouts).

Shortly following each trial, the participants were asked to answer an agreement rating of six forms of questions on a 7-point Likert scale. This ranged from 0 (completely disagree) to 6 (completely agree), where Q1 (ownership), Q2 (numbness), Q3 (longer deformation), and Q4 (wider deformation) are illusion statements, while Q5 (pins and needles) and Q6 (smaller deformation) are control statements (See the detail in Figure 3). Furthermore, the order of the questions was determined in a-pseudo-randomized manner for each participant. All participants experienced 20 sessions (2 layout conditions × 5 distance conditions × 2), where each of the two layout conditions was performed alternately. In addition, the order of the five distance conditions was determined at random (Figure 2B).

1.3 Data Analysis

A two-way within-participants ANOVA was applied for the layout and distance factors. If the assumption of sphericity was violated (indicated by Mendoza's Multi-sample Test for Sphericity), we used Greenhouse-Geisser's epsilon to correct the degrees of freedom. A simple main effect test was conducted on the obtained interactions (layout vs. distance), and post-hoc tests were performed using Holm's modified sequentially rejective Bonferroni procedure. The statistical analyses used R version 4.1.1 (R Core Team 2021) and the anovakun version 4.8.5 function in R.

1.4 Results

A two-way within-participant ANOVA (layout × distance) revealed a significant main effect of the layout in ownership (Q1: $F(1, 25) = 6.73$, $p = .02$, $\eta_p^2 = .21$), longer deformation (Q3: $F(1, 25) = 38.0$, $p < .001$, $\eta_p^2 = .60$), and wider deformation (Q4: $F(1, 25) = 18.1$, $p < .001$, $\eta_p^2 = .42$), but not in numbness (Q2: $F(1, 25) = 3.12$, $p = .38$, $\eta_p^2 = .03$) and the control statements of Q5 and Q6. The analysis also found significant main effect of distance in ownership(Q1: $F(4, 100) = 5.50$, $p = .005$, $\eta_p^2 = .18$), numbness(Q2: $F(4, 100) = 3.12$, $p = .02$, $\eta_p^2 = .11$), and wider deformation (Q4: $F(4, 100) = 12.00$, $p < .001$, $\eta_p^2 = .32$), but not in longer deformation (Q3: $F(4, 100) = 2.70$, $p = .06$, $\eta_p^2 = .10$) and the control statements of Q5 and Q6. In addition, it revealed significant interactions between the two factors in ownership (Q1: $F(4, 100) = 5.11$, $p = .008$, $\eta_p^2 = .17$) and longer deformation (Q3: $F(4, 100) = 4.50$, $p = .01$, $\eta_p^2 = .15$), wider deformation (Q4: $F(4, 100) = 7.46$, $p = .001$, $\eta_p^2 = .23$), but not numbness (Q2: $F(4, 100) = 2.30$, $p = .06$, $\eta_p^2 = .08$) and the control statements of Q5 and Q6.

Consequently, a follow-up test with a simple effect analysis and multiple comparisons was examined. For the ownership statement (Q1), the layout had a significant simple effect under the 3–6 cm distance conditions (3 cm: $F(1, 25) = 5.98$, $p = .02$, $\eta_p^2 = .19$; 4 cm: $F(1, 25) = 8.21$, $p = .008$, $\eta_p^2 = .25$; 5 cm: $F(1, 20) = 8.71$, $p = .007$, $\eta_p^2 = .26$; 6 cm: $F(1, 25) = 7.70$, $p = .01$, $\eta_p^2 = .24$), but not 2 cm ($F(1, 25) = 7.70$, $p = .47$, $\eta_p^2 = .02$). Thus, when the double-touch distance is 3 cm above, sense of ownership was rated higher for the distal layout compared to the lateral layout. In addition, distance had a significant simple effect under the lateral layout ($F(4, 100) = 9.51$, $p < .001$, $\eta_p^2 = .28$) but not under the distal layout ($F(4, 100) = 2.03$, $p = .12$, $\eta_p^2 = .08$). Specifically, under the lateral layout, the ownership rating at a distance of 2 cm was significantly higher than 4–6 cm (2 cm vs. 4 cm: $p < .05$; 2 cm vs. 5 cm: $p < .05$; 2 cm vs. 6 cm: $p < .01$), and that of 3 cm was significantly higher than 6 cm (3 cm vs. 6 cm: $p < .05$). Furthermore, for the statement of longer deformation (Q3), the layout had a significant simple effect for all distance

conditions (2 cm: $F(1, 25) = 14.6$, $p < .001$, $\eta_p^2 = .37$; 3 cm: $F(1, 25) = 36.9$, $p < .001$, $\eta_p^2 = .60$; 4 cm: $F(1, 25) = 32.0$, $p < .001$, $\eta_p^2 = .56$; 5 cm: $F(1, 25) = 35.0$, $p < .001$, $\eta_p^2 = .58$; 6 cm: $F(1, 25) = 21.2$, $p < .001$, $\eta_p^2 = .46$), and distance had a significant simple effect under the distal layout condition ($F(4, 100) = 3.64$, $p = .03$, $\eta_p^2 = .13$) but not under the lateral layout condition($F(4, 100) = 1.07$, $p = .37$, $\eta_p^2 = .04$). Under the distal layout condition, the longer deformation rating at a distance of 2 cm was significantly below 3–4 cm (2 cm vs. 3 cm: $p < .05$; 2 cm vs. 4 cm: $p < .01$). For the statement of wider deformation (Q4), the layout had a significant simple effect for all distance conditions (2 cm: $F(1, 25) = 19.9$, $p < .001$, $\eta_p^2 = .44$; 3 cm: $F(1, 25) = 17.0$, $p < .001$, $\eta_p^2 = .40$; 4 cm: $F(1, 25) = 7.4$, $p = .01$, $\eta_p^2 = .23$; 5 cm: $F(1, 25) = 6.02$, $p = .02$, $\eta_p^2 = .19$; 6 cm : $F(1, 25) = 7.78$, $p = .01$, $\eta_p^2 = .24$）. In addition, distance had a significant simple effect under the lateral layout condition ($F(4, 100) = 11.0$, $p < .001$, $\eta_p^2 = .30$) but not under the distal condition ($F(4, 100) = 2.27$, $p = .11$, $\eta_p^2 = .08$). Under the lateral layout condition, the longer deformation rating at a distance of 2 cm was significantly higher than 4–6 cm ($p < .01$, for all), and that of 3 cm was significantly higher than 4–6 cm ($p < .05$, for all）.

1.5 Discussion

The results indicate that a synchronized double-touch of both one's own finger and another person's finger leads to the extension of ownership through the other person's finger, involving the sense of the finger's extended deformation, and an illusory finger deformation is more likely to occur in cases of length deformation than in cases of width deformation. The reasons for this are the following. First, significantly higher ownership ratings were found in the distal layout than in the lateral layout in the 3, 4, 5, and 6 cm conditions, indicating that the effects of double-touch and the sense of ownership are more easily induced in the direction of the finger length than in the width direction. Second, a deformable boundary based on points of significantly declining ownership ratings was found between 2 cm and 4 cm in the lateral layout, but this was not found in the distal layout. This indicates a difference in the boundary of illusory finger deformation between the length and width of the finger, with the boundary showing greater length than width.

References

Campbell, J. I. D., & Thompson, V. A. (2012). MorePower 6.0 for ANOVA with relational confidence intervals and Bayesian analysis. *Behavior Research Methods*, *44*(4), 1255–1265. https://doi.org/10.3758/s13428-012-0186-0

Okubo M., Suzuki H., & Nicholls M. E. R. (2014). A Japanese version of the FLANDERS handedness questionnaire. *The Japanese journal of psychology*, *85*(5), 474–481. https://doi.org/10.4992/jjpsy.85.13235

R Core Team (2021). R: A language and environment for statistical computing. R Foundation for Statistical Computing, Vienna, Austria. URL https://www.R-project.org/.

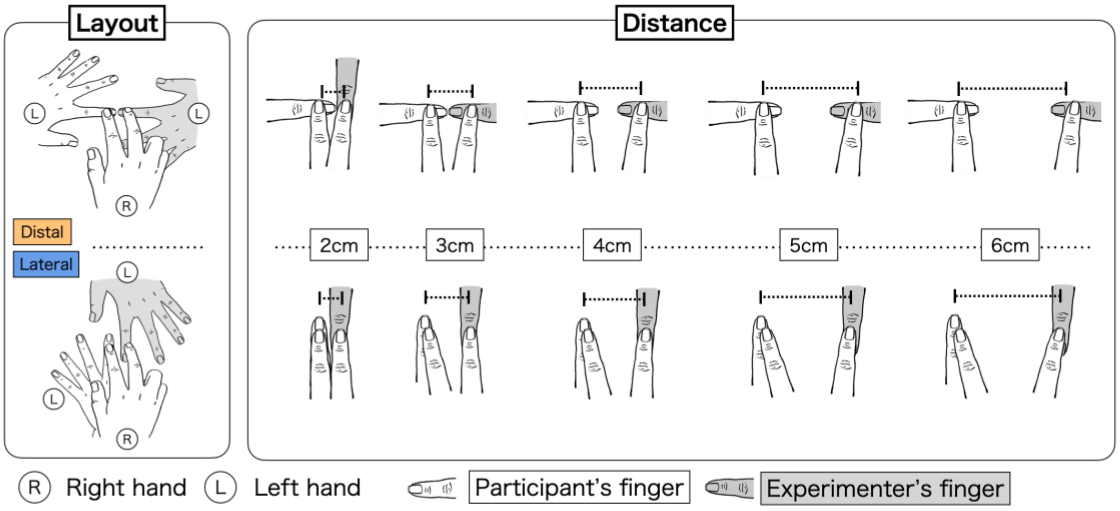

Figure 1. There are two layout and five distance conditions. The double touch operation was asked to provide around the first joint of the two receptive fingers and not to touch their fingernails. In the 2 cm distal layout, to prevent mutual overlap, the experimenter's left index finger was replaced with the little finger placed orthogonally to the participant's index finger.

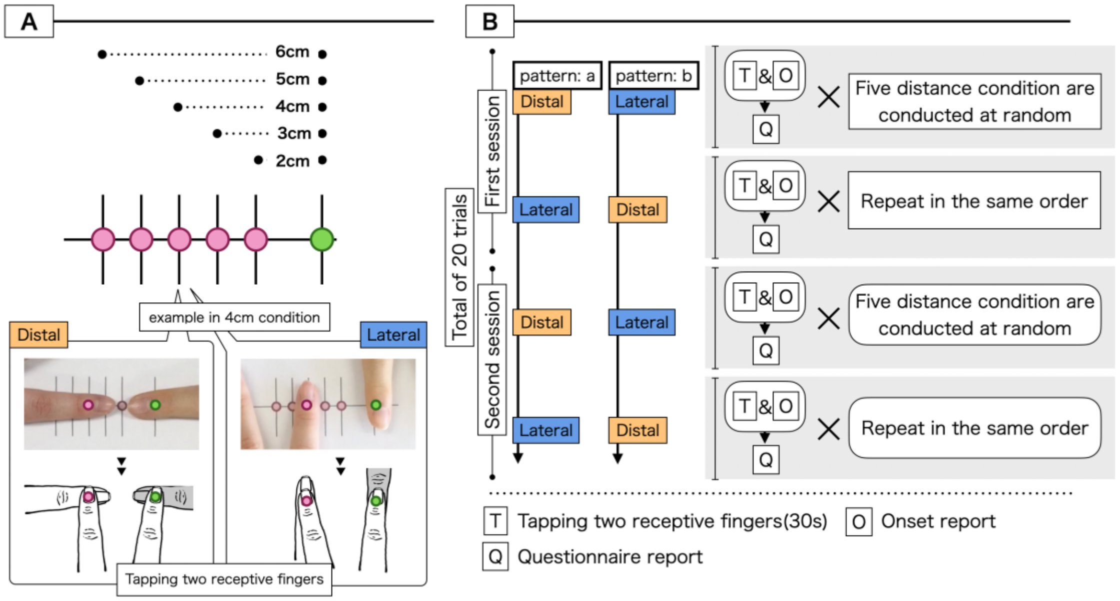

Figure 2: (A) highlights the way of setting the double-touch layout in each distance layout using a sheet printing six circle-shaped guides indicating the position where the participant should tap. The pink circle corresponds to the touch to the self-receptive finger, while the green circle corresponds to the touch to the other's receptive finger. (B) shows the diagram of the experimental procedure containing a total of 20 trials. The onset report was exhibited during the 30-sec tapping trial following the questionnaire's response. Two patterns of the layout condition order (distal to lateral or lateral to distal) were allocated to participants in a counter-balanced manner.

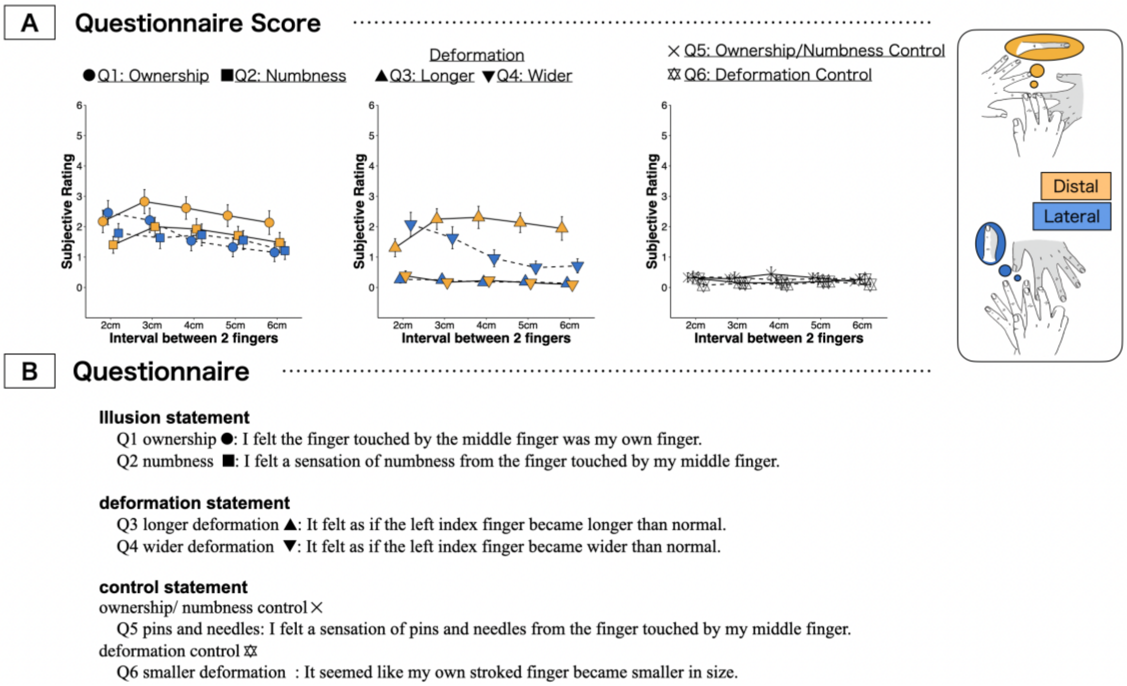

B Questionnaire

Illusion statement

Q1 ownership ●: I felt the finger touched by the middle finger was my own finger.

Q2 numbness ■: I felt a sensation of numbness from the finger touched by my middle finger.

deformation statement

Q3 longer deformation ▲: It felt as if the left index finger became longer than normal.

Q4 wider deformation ▼: It felt as if the left index finger became wider than normal.

control statement

ownership/ numbness control ×

Q5 pins and needles: I felt a sensation of pins and needles from the finger touched by my middle finger.

deformation control ✡

Q6 smaller deformation : It seemed like my own stroked finger became smaller in size.

Figure 3: (A) depicts the average of the questionnaire's agreement rating for ownership, numbness, and deformation (Longer finger/ Wider finger), in addition, each control statements. Error bars indicate standard error.
